# The composition of the lung microbiome differs between patients with dermatomyositis and rheumatoid arthritis associated with interstitial lung disease

**DOI:** 10.1002/2211-5463.13334

**Published:** 2021-12-18

**Authors:** Yueyan Lou, Qing Wei, Bijun Fan, Liyan Zhang, Xiaodong Wang, Zhiwei Chen, Xiaoming Tan, Yu Zheng

**Affiliations:** ^1^ Department of Pulmonology Renji Hospital Shanghai Jiaotong University School of Medicine Shanghai China; ^2^ Department of Laboratory Medicine Renji Hospital Shanghai Jiaotong University School of Medicine Shanghai China; ^3^ Department of Rheumatology Renji Hospital Shanghai Jiaotong University School of Medicine Shanghai China

**Keywords:** bronchoalveolar lavage fluid, dermatomyositis, interstitial lung disease, microbiome, rheumatoid arthritis

## Abstract

Dermatomyositis and rheumatoid arthritis are inflammatory diseases that affect the skeletal muscles and joints, respectively. A common systemic complication of these diseases is interstitial lung disease (ILD), which leads to a poor prognosis and increased mortality. However, the mechanism for the initiation and development of ILD in patients with dermatomyositis is currently unknown. In the present study, we used 16S rRNA high‐throughput sequencing to profile the bacterial community composition of bronchoalveolar lavage fluid of patients with dermatomyositis associated with ILD (DM‐ILD; shortened to DM below), rheumatoid arthritis associated with ILD (RA‐ILD; shortened to RA below) and healthy controls (N) aiming to understand the differences in their lung microbiota and to predict gene function. We found that there were more operational taxonomic units (OTUs) in the lung microbiota of both RA and DM compared to N, although there was no significant difference in the number of OTUs between RA and DM. Similarly, the diversity in alphaproteobacteria differed between RA and DM compared to N, but not between RA and DM. The lung microbiota of RA, DM and N was mainly comprised of five phyla: Firmicutes, Bacteroidetes, Proteobacteria, Actinobacteria and Fusobacteria, with 10 dominant genera. Despite the similarity in microbiota composition, we also identified 41 OTUs of lung microbiota that differed among RA, DM and N. Additionally, linear discriminant analysis effect size and linear discriminant analysis genus scores confirmed that 31 microbial biomarkers were clearly distinguished among RA, DM and N. The functional and metabolic alterations of the lung microbiota among RA, DM and N were predicted using picrust, and differentially abundant KEGG (Kyoto Encyclopedia of Genes and Genomes) pathways were identified. Research on the lung microbiota of patients with DM and RA may open new opportunities for developing biomarkers to identify high‐risk patients.

## Graphical abstract

Dermatomyositis (DM) and rheumatoid arthritis (RA) are inflammatory diseases. Interstitial lung disease (ILD) is a common complication of these conditions and leads to poor prognosis and higher mortality. The initiation and development of ILD in patients with DM and RA is poorly understood. Using 16S rRNA high‐throughput sequencing, we described the lung microbiota of patients with DM‐ and RA‐associated ILD and compared it with that of healthy individuals.

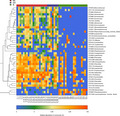

**Abbreviations**
ACE: angiotensin‐converting enzyme index
BALF: bronchoalveolar lavage fluid
CRP: C‐reactive protein
DM: dermatomyositis
DM‐ILD: dermatomyositis associated with ILD
ESR: erythrocyte sedimentation rate
IL: interleukin
ILD: interstitial lung disease
KEGG: Kyoto Encyclopedia of Genes and Genomes
LDA: linear discriminant analysis
LEfSe: linear discriminant analysis effect size
N: healthy controls
OTUs: operational taxonomic units
PC: principal component
PCoA: principal coordinate analysis
RA: rheumatoid arthritis
RA‐ILD: rheumatoid arthritis associated with ILD

Dermatomyositis [1] is a type of idiopathic inflammatory myopathy that is characterized by the inflammation of the skeletal muscle and skin and involves a characteristic heliotrope skin rash and Gottron’s papules [2]. Interstitial lung disease (ILD) is considered a common systemic complication of DM [3]. DM associated with ILD (DM‐ILD) is one of the major extra muscular manifestations contributing to increased morbidity and mortality [4]. ILD is also one of the life‐threatening complications of clinically amyopathic dermatomyositis. The overall prognosis is grave, with a 33%–67% 6‐month mortality despite aggressive immunosuppressive therapy [5, 6, 7, 8, 9]. However, the mechanism of the initiation and development of disease progression in DM‐ILD is not understood.

Subsequent to the first culture‐independent report of the microbiota in the lower respiratory tract [10], accumulating evidence supports potential functional roles for the lung microbiota [11, 12]. Dysbiosis of the lung microbiota can underlie lung diseases such as cystic fibrosis [13], asthma [10], chronic obstructive pulmonary diseases [14], bronchiectasis [15], sarcoidosis [16] or even lung cancer [17]. Recently, emerging evidence has shown that the increased bacterial burden of potentially pathogenic bacteria may lead to disease progression in idiopathic pulmonary fibrosis [18, 19]. We are also now beginning to understand the composition of the lung microbiota in other ILDs associated with rheumatoid arthritis (RA) [20]. However, the potential contribution of the lung microbiome in DM‐ILD has never been investigated.

The present study aimed to characterize the lung microbiota in patients with DM‐ILD (DM group) and compare its composition and diversity with the results obtained from patients with RA (RA group) and historic healthy controls (N group).

## Materials and methods

### Participant recruitment and sample collection

The study was approved by the Ethics Committee of Renji Hospital, School of Medicine, Shanghai Jiaotong University (2016075) and written informed consent was obtained from all participants. The study methodologies conformed to the standards set by the Declaration of Helsinki. All participants consented to a bronchoscopy examination at Renji Hospital. Medical history was collected from all participants and a set of routine pre‐procedure tests, including physical examination, electrocardiogram, pulmonary function testing, computed tomography, routine blood count and blood coagulation function analysis, were carried out. Patients in the DM group were diagnosed with dermatomyositis in accordance with the classification criteria proposed by Bohan and Peter [21, 22]. Patients in the RA group were diagnosed with rheumatoid arthritis according to the 1987 ACR criteria [23]. The diagnosis of interstitial lung disease was based on respiratory symptoms and the presence of bibasilar infiltrates on high‐resolution computed tomography. The criteria for selecting healthy control participants were: good physical status, no significant respiratory conditions, and normal findings on pre‐procedure examinations and bronchoscopy. The study methodologies conformed to the standards set by the Declaration of Helsinki.

Bronchoscopy was performed as previously described [24]. Bronchoscopy via the nasal route was performed to obtain bronchoalveolar lavage fluid (BALF) samples from patients in the RA and DM groups and one BALF sample from each control. All samples were immediately frozen and maintained at −80 °C until further DNA extraction.

### DNA extraction, 16S rRNA amplification and sequencing

All BALF samples were subjected to the same procedures for DNA extraction and PCR amplification by the same laboratory staff. The BALF sample was centrifuged at 17 465 ***g*** for 30 min at 4 °C, and then, the pellet was suspended in 790 μL of sterile lysis buffer (4 m guanidine thiocyanate; 10% *N*‐lauroyl sarcosine; 5% *N*‐lauroyl sarcosine‐0.1 m phosphate buffer, pH 8.0) in a 2‐mL screw‐cap tube containing 1 g of glass beads (0.1 mm; BioSpec Products Inc., Bartlesville, OK, USA). This mixture was vortexed vigorously and then incubated at 70 °C for 1 h. After incubation using bead beating for 10 min at maximum speed, DNA was extracted in accordance with the manufacturer’s instructions for bacterial DNA extraction using The E.Z.N.A.®Stool DNA Kit (Omega Bio‐tek Inc., Norcross GA, USA), with the exception of the lysis steps, and stored at −20 °C for further analysis. The blank reagents were set as a negative control to avoid contamination in the extraction progress. The extracted DNA from each sample and the negative control were used as the template to amplify the V3–V4 region of 16S rRNA genes.

The primers F1 and R2 (5′‐CCTACGGGNGGCWGCAG‐3′ and 5′‐GACTACHVGGGTATCTAATCC‐3′) corresponding to positions 341–805 in the *Escherichia coli* 16S rRNA gene were used to amplify the V3–V4 region of each BALF sample by PCR. The PCR reagents were set as a negative control for the PCR step. PCR reactions were run in an EasyCycler 96 PCR system (Analytik Jena Corp., Jena, Germany) using the program: 2 min of denaturation at 95 °C, followed by 25 cycles for 30 s at 95 °C (denaturation), 30 s for annealing at 55 °C and 30 s at 72 °C (elongation), with a final extension at 72 °C for 5 min. The products from different samples were indexed and mixed at equal ratios for sequencing by Shanghai Mobio Biomedical Technology Co. Ltd (Shanghai, China) using the Miseq platform (Illumina Inc., San Diego, CA, USA) in accordance with the manufacturer’s instructions.

### Bioinformatics analysis for sequencing data

Clean data were extracted from the raw data using usearch, version 8.0 with the following criteria: (a) Sequences of each sample were extracted using each index with zero mismatches; (b) sequences with an overlap of < 50 bp were discarded; [18] the error rate of overlap > 0.1 was discarded; and (c) sequences < 400 bp after merge were discarded. Operational taxonomic units (OTUs) were classified based on 97% similarity after chimeric sequences were removed using uparse, version 7.1 (http://drive 5.com/uparse). The phylogenetic profile of each 16S rRNA gene sequence was analyzed via rdp classifier (http://rdp.cme.msu.edu) against the Silva (SSU123) 16S rRNA database with a confidence threshold of 70%.

Sample diversity metrics were assessed based on the non‐parametric Shannon–Wiener diversity index, Chao index and abundance‐based coverage estimators index [25]. The Bray–Curtis distance was calculated in qiime (http://qiime.org). The qiime pipeline was also used to generate principal coordinate analysis (PCoA) plots to visualize Bray–Curtis dissimilarity. The Mann–Whitney test and Kruskal–Wallis rank sum test were used to test for statistical significance between and among the bacterial types in different groups (qiime package). Adonis analysis was used to estimate the significance between different groups. The linear discriminant analysis effect size (LEfSe) was used to detect taxa with differential abundance among groups. Bar plots, PCoA plots and Venn diagrams were all generated in r (http://www.R‐project.org).

The Phylogenetic Investigation of Communities by Reconstruction of Unobserved States (picrust) (http://picrust.github.io/picrust) predicts the metabolic functions of bacterial flora and 16S rRNA gene sequences in the Kyoto Encyclopedia of Genes and Genomes (KEGG). picrust recaptures key findings from the Human Microbiome Project by an extended ancestral state reconstruction algorithm and accurately predicts the abundance in host‐associated communities of the gene families, with quantifiable uncertainty.

Raw sequencing data of the 16S rRNA gene V3–V4 regions and accompanying information are available in the Sequence Read Archive database (https://www.ncbi.nlm.nih.gov/sra) under accession number PRJNA714758 (RA, DM) and SRP110884 (healthy controls).

## Results

**Fig. 1:**
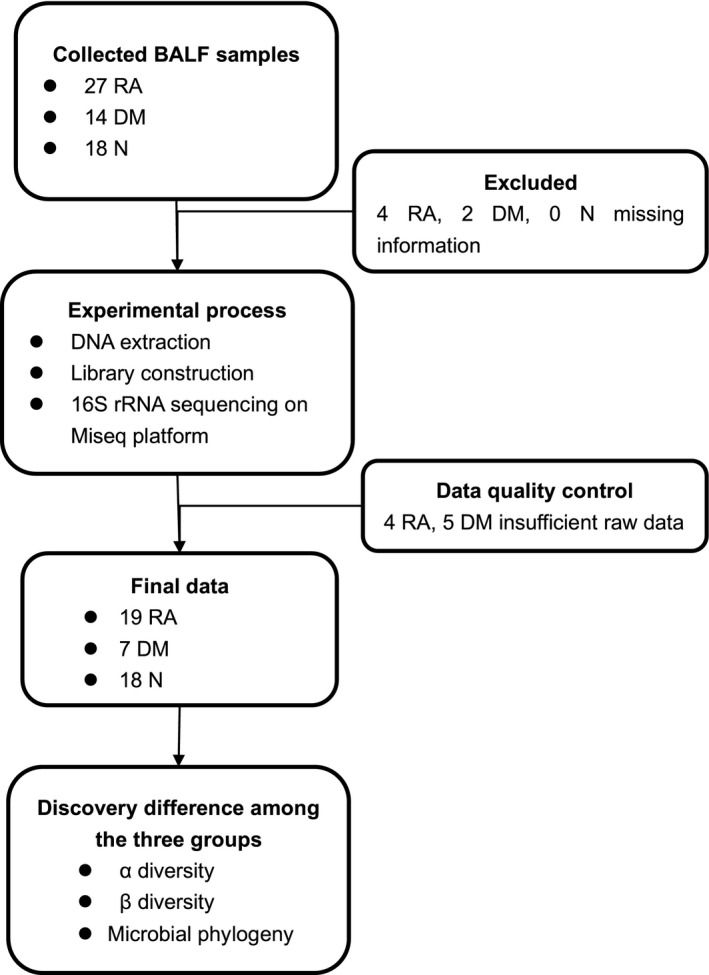
Study design and flow diagram. In total, 59 BALF samples were collected prospectively. After rigorous diagnostic and exclusion procedures, 46 samples were included for analysis, including 19 RA, seven DM and 18 N. In this discovery cohort, we used 16S rRNA high‐throughput sequencing to characterize the lung microbiota between RA, DM, and N.

In total, 59 BALF samples were prospectively collected. After rigorous diagnosis and exclusion procedures, 46 samples were included for analysis, including 19 RA, seven DM and 18 N from the Renji Hospital. All samples that belonged to different groups were used to characterize the differences in lung microbiota of the study participants (Fig. 1).

### Baseline characteristics

The present study included 18 healthy controls, seven patients with DM‐ILD and 19 patients with RA‐ILD. Serological profiling of each patient, including C‐reactive protein (CRP), erythrocyte sedimentation rate (ESR), interleukin (IL)‐2 receptor, IL‐6 and IL‐8, was performed using standard methods. The clinical characteristics are presented in Table 1.

**Table 1:** Demographics and clinical and functional parameters of the DM‐ILD, RA‐ILD and healthy control groups.

Parameters	RA‐ILD	DM‐ILD	Healthy controls	Pooled analysis	Test
	*n* = 19	*n* = 7	*n* = 18	*P‐*value	
Age (years)	45.84 (15.52)	52.78 (13.13)	36.83 (11.51)	0.709	KW
Sex (female/male)^*^	11/8	4/3	7/11	0.035	KW
Smoking				0.381	KW
Non‐smokers, *n* (%)	11 (57.9%)	4 (57.1%)	13 (72.2%)		
Ex‐smokers, *n* (%)	6 (31.6%)	3 (42.9%)	3 (16.7%)		
Current‐smokers, *n* (%)	2 (10.5%)	2 (28.6%)	2 (11.1%)		
CRP (mg·L^−1^)	6.84 (6.59)	9.25 (1.32)	NA	0.119	KW
ESR (mm·H^−1^)	14.89 (11.83)	27.25 (26.61)	NA	0.578	KW
IL‐2 receptor (U·mL^−1^)	667.75(551.42)	737.33 (219.59)	NA	0.160	KW
IL‐6 (pg·mL^−1^)	12.20 (22.32)	4.86 (3.70)	NA	0.386	KW
IL‐8 (pg·mL^−1^)	123.38 (102.00)	72.48 (38.42)	NA	0.291	KW
Pulmonary function test					
FVC^*^	2.11 (0.81)	2.50 (0.78)	3.27 (0.92)	0.001	ANOVA
FVC (% predicted)^*^	68.47 (17.62)	78.03 (12.54)	98.77 (11.05)	0.0001	ANOVA
FEV1 (% predicted)^*^	69.41 (19.14)	80.82 (13.13)	100.32 (9.81)	0.0001	ANOVA
DLCO (% predicted)^*^	43.55 (17.28)	42.74 (10.80)	70.55 (12.45)	0.0001	
Bronchoscopy					
Macrophages	39.44 (33.50)	65.33 (31.81)	147.88 (39.07)	0.243	ANOVA
Lymphocytes	8.22 (10.23)	8.71 (10.55)	4.81 (5.97)	0.716	KW
Neutrophils	41.22 (31.08)	21.56 (26.64)	28.50 (31.37)	0.213	ANOVA
Eosinophils	0 (0‐0)	0.17 (0.41)	0 (0‐0)	0.055	ANOVA

Age, CRP, ESR, FVC, FVC % predicted, FEV1% predicted, DLCO % predicted, IL‐2, IL‐6, IL‐8, BALF macrophages, lymphocytes, neutrophils and eosinophils are expressed as the mean (SD). Between‐group comparisons were made with one‐way analysis of variance (ANOVA) for normally distributed continuous variables and with the Kruskal–Wallis (KW) test for non‐normally distributed continuous variables. *P < 0.05. FVC, forced vital capacity; DLCO, diffusing capacity of carbon monoxide; NA, not available.

### Characteristics of 16S rRNA gene sequencing in BALF samples

Bacterial 16S rRNAs were detected in all BALF specimens. Both negative controls (blank reagents) from the extraction and PCR steps showed no bands in the agarose gel; thus, all of the PCR products were from the samples, and there was no contamination from reagents or the environment.

Over 1 692 237 high‐quality sequencing reads were obtained from the 46 specimens, with an average of 36 787.8 per sample. The Good’s coverage of each sample was > 99.90%, indicating that the sequencing reads identified represent the majority of bacteria in each of the BALF samples.

### The diversity of lung microbiota among RA, DM and N

**Fig. 2:**
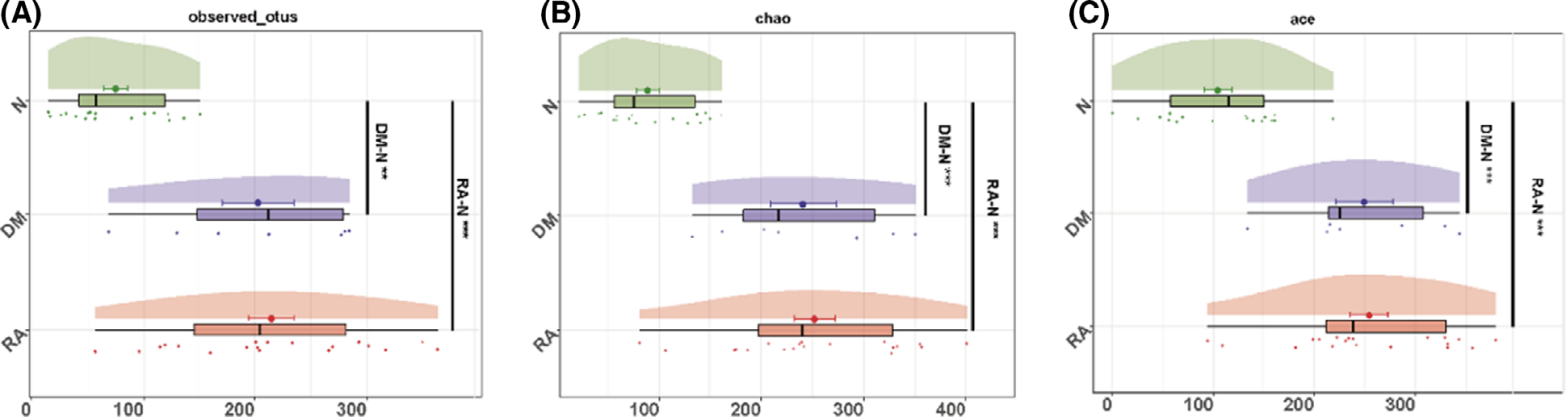
The diversity of lung microbiota in RA (*n* = 19), DM (*n* = 7) and N (*n* = 18) at the OTU level. (A) Observed OTUs in RA (red) DM (purple) and N (green). (B) Chao index in RA (red), DM (purple) and N (green). (C) ACE index in RA (red), DM (purple) and N (green). A Mann–Whitney test was used to determine the significance between RA and DM, RA and N, and DM and N. **P < 0.01; ***P < 0.001.

The mean ± SE number of OTUs was 214.84 ± 20.20 in the BALF samples from the RA group, which was higher than that of the N group (75.83 ± 10.47, *P* < 0.0001). There were 202.86 ± 31.80 OTUs in the BALF samples from the DM group, which was higher than that of the N group (75.83 ± 10.47, *P* = 0.001). However, there was no significant difference in the OTUs of BALF samples between the RA and DM groups (*P* = 0.82). Among the three groups, the angiotensin‐converting enzyme (ACE) index was higher in RA than in N (254.49 ± 18.44 vs. 104.37 ± 13.97, *P* < 0.001) and it was higher in DM than in N (249.71 ± 28.03 vs. 104.37 ± 13.97, *P* < 0.001). There were no significant differences in the ACE index between the RA and DM groups (*P* = 0.65). The Chao indices were also higher in RA than in N (202.20 ± 20.01 vs. 88.65 ± 10.77, *P* < 0.001) and higher in DM than in N (240.96 ± 31.76 vs. 88.65 ± 10.77, *P* < 0.001), with no significant difference between the RA and DM groups (*P* = 0.65) (Fig. 2). The Shannon index was not significantly different among RA vs. N (2.93 vs. 2.91, *P* = 0.44), DM vs. N (2.34 vs. 2.91, *P* = 0.11) and RA vs. DM (2.93 vs. 2.34, *P* = 0.21).

**Fig. 3:**
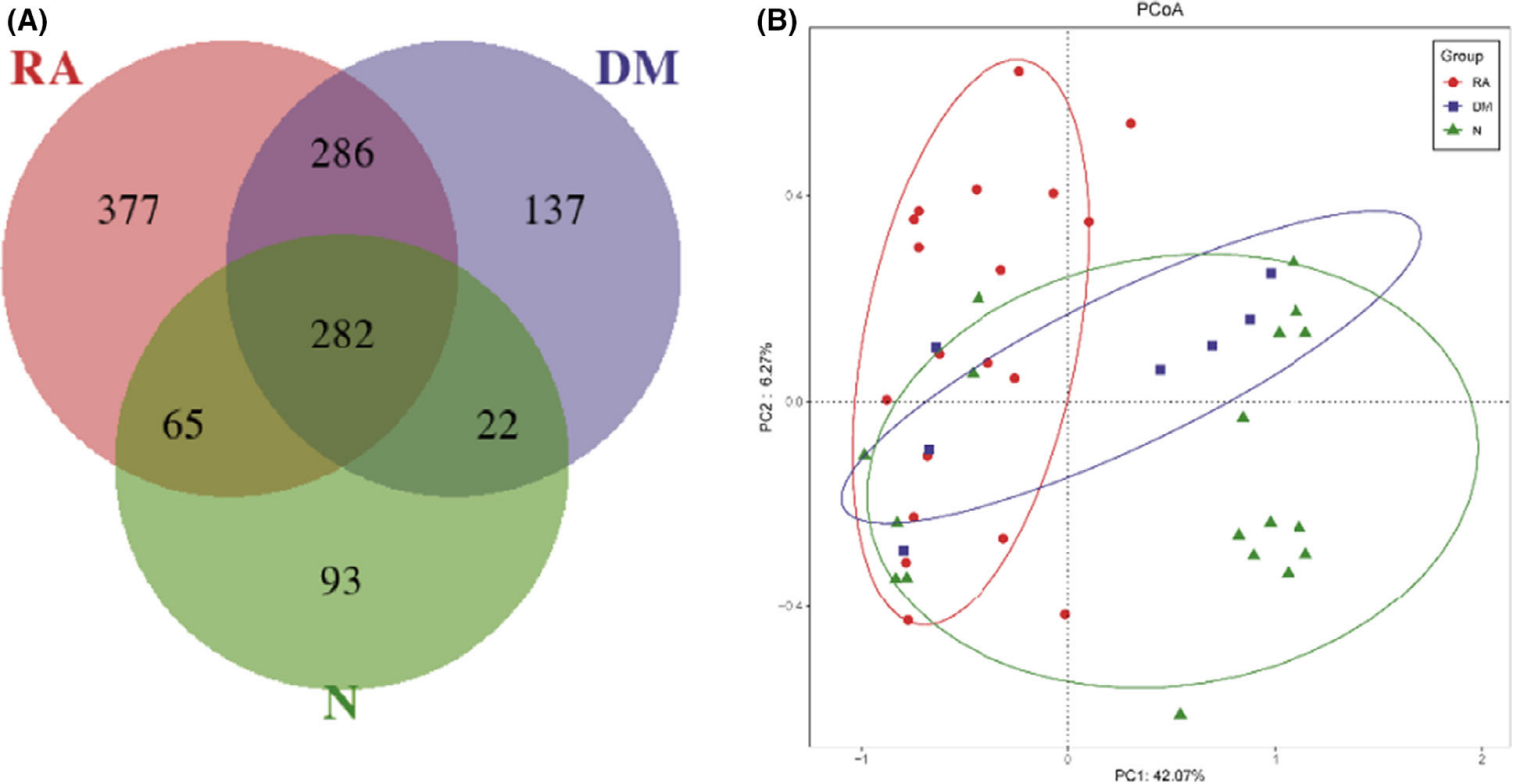
Comparison of the differences in lung microbiota. (A) A Venn diagram displaying overlaps among groups showed that 282 of the 1262 OTUs were common to all the groups, whereas 377 were unique to RA and 137 were unique to DM. (B) Lung microbiota structure in RA (*n* = 19), DM (*n* = 7) and N (*n* = 18) using PCoA based on the Bray–Curtis distance. PCoA of the Bray–Curtis distance PC1–2 showed that the samples of the RA (red), DM (blue) and N (green) groups were distinctly separated in the direction of the PC2 axis, which means that the overall fecal microbiota compositions were markedly different among RA, DM and N. Each symbol represents a sample (red, RA; blue, DM; green, N). Variance explained by the PCs is indicated in the parentheses on the axes.

A Venn diagram showed that 282 of the 1262 OTUs were common to all the groups, whereas 377 were unique to RA and 137 were unique to DM (Fig. 3A). Notably, compared to the N group, there were many more OTUs increased in RA and DM, implying that there were microbial differences among RA, DM and N.

To compare the overall lung microbiota composition among the three groups, PCoA analysis based on the Bray–Curtis distance and weighted unique fraction distance according to the OTUs of each sample was conducted. Overall, in the total variation of all BALF samples, the first principal component (PC1) could account for 42.07% and PC2 could account for 6.27%. As shown in Fig. 3B, the PCoA revealed a difference among RA, DM and N with respect to lung bacterial composition (*P* = 0.0015, Adonis analysis).

### The composition of lung microbiota in RA, DM and N

**Fig. 4:**
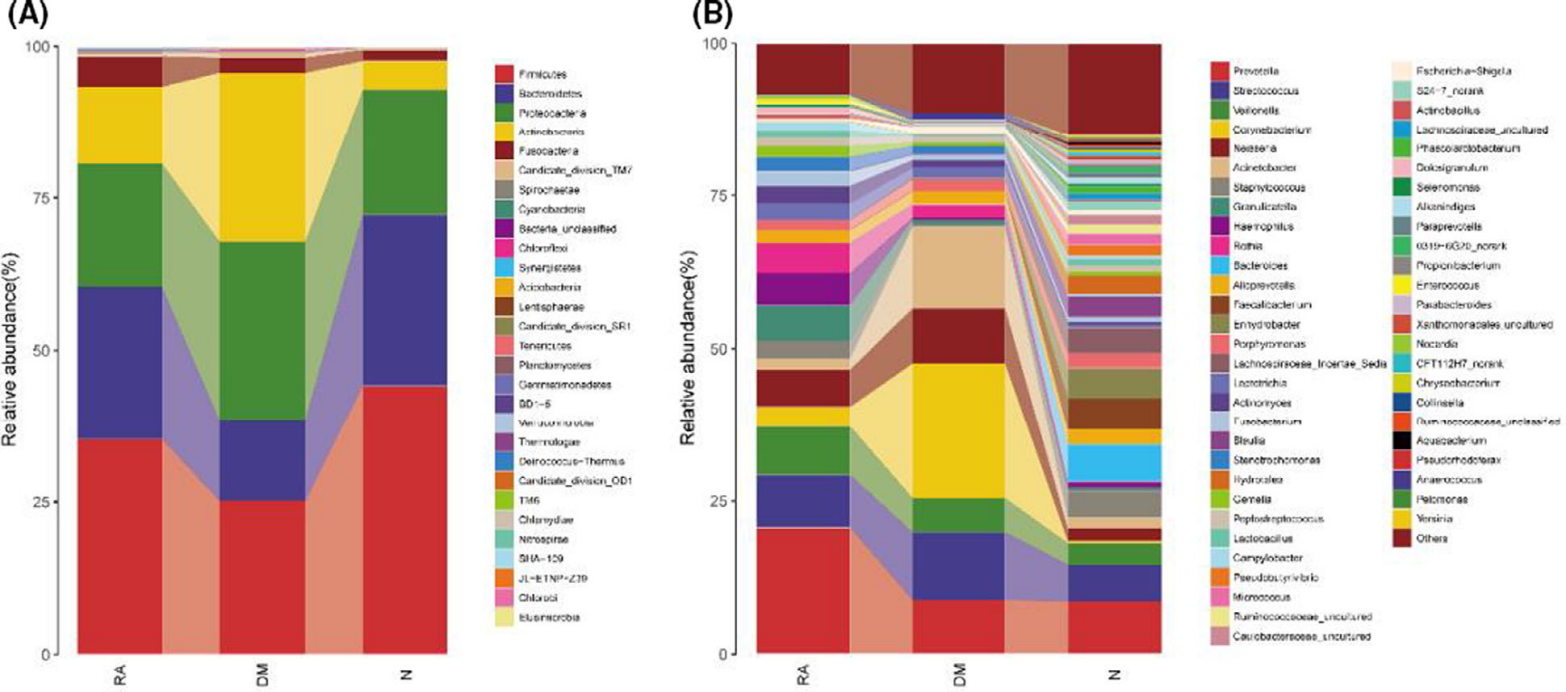
Composition and comparison of lung microbiota in RA (*n* = 19), DM (*n* = 7) and N (*n* = 18). Composition of lung microbiota at the (A) phylum and (B) genus levels.

At the phylum level, the lung microbiota mainly comprised five phyla (Fig. 4A); at the genus level, the lung microbiota mainly comprised 10 dominant genera (Fig. 4B); and the relative abundances for each phylum were comparable in RA, DM and N (mean ± SE) (Table 2).

**Table 2:** Relative abundances (%) of lung microbiota at the phylum and genus levels in RA, DM, and healthy controls. A Kruskal–Wallis rank sum test was used for analysis the significance among RA, DM and N.

	RA (%) (mean ± SE)	DM (%) (mean ± SE)	N (%) (mean ± SE)	*P*‐value
Phylum level				
Firmicutes	35.35 ± 2.90	25.24 ± 9.51	44.25 ± 4.57	0.0562
Bacteroidetes	25.08 ± 3.85	13.34 ± 5.18	28.03 ± 3.19	0.0925
Proteobacteria	20.21 ± 3.36	29.32 ± 14.75	20.65 ± 5.62	0.5985
Actinobacteria^*^	12.75 ± 2.84	27.78 ± 13.50	4.69 ± 1.75	0.0037
Fusobacteria^*^	5.04 ± 1.01	2.48 ± 0.81	1.84 ± 0.68	0.0162
Genus level				
Prevotella^*^	20.64 ± 3.96	8.86 ± 3.76	8.63 ± 2.46	0.0258
Streptococcus	8.58 ± 1.41	11.03 ± 6.43	5.96 ± 2.10	0.1248
Veillonella^*^	8.00 ± 1.28	5.58 ± 2.79	3.54 ± 1.15	0.0156
Corynebacterium^*^	3.26 ± 1.72	22.14 ± 14.31	0.39 ± 0.34	0.0008
Neisseria	6.12 ± 2.15	8.95 ± 7.91	2.03 ± 0.86	0.1394
Acinetobacter	1.78 ± 1.36	13.29 ± 12.78	1.76 ± 0.76	0.3251
Staphylococcus	2.92 ± 1.86	0.55 ± 0.35	4.3 ± 3.85	0.1846
Granulicatella^*^	5.78 ± 2.08	0.63 ± 0.4	0.52 ± 0.23	0.0002
Haemophilus^*^	5.25 ± 2.03	0.37 ± 0.21	0.83 ± 0.32	0.0251
Rothia^*^	4.92 ± 2.56	2.13 ± 1.6	0.23 ± 0.09	0.0002

The data are expressed as the mean ± SE. Between‐groups comparisons were made with one‐way analysis of variance for normally distributed continuous variables. *P < 0.05.

**Fig. 5:**
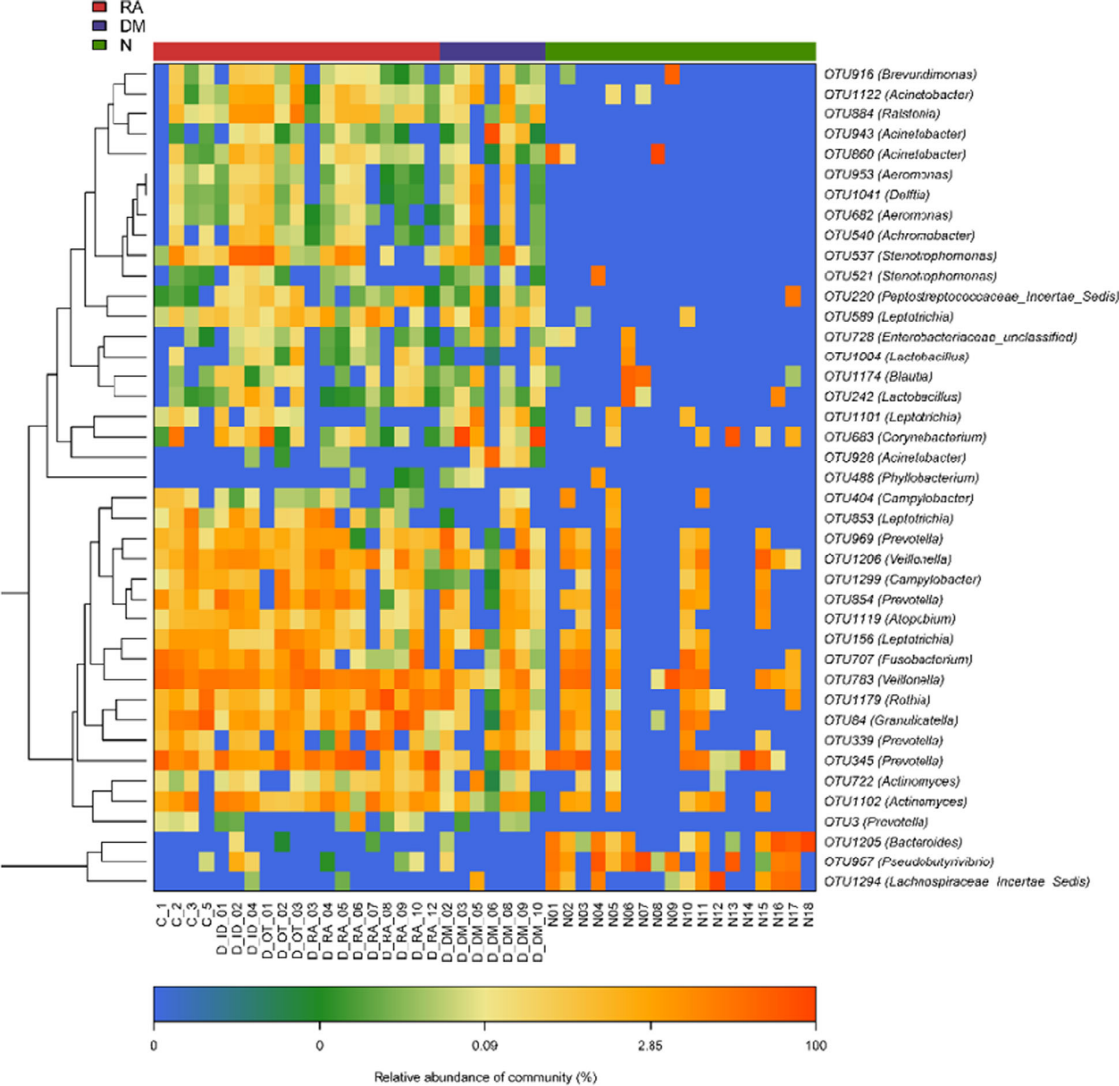
Heatmaps for the relative abundances of differential OTUs among RA (*n* = 19), DM (*n* = 7) and N (*n* = 18). For each sample, the columns show a relative abundance of data for differential OTUs on the right. The relative abundance of each OTU was used to plot the heatmap (blue, low abundance; red, high abundance). Group data are shown above the plot: RA, left, red line; DM, middle, blue line; N, right, green line. Each row represents one OTU. A random forest model was used for screening the differential OTUs.

Despite the above similarity, the present study also identified 41 OTUs of lung microbiota that differed among RA, DM and N (Fig. 5 and Table 2). The relative abundances of *Bacteroides* (OTU 1205), *Prevotella* (OTU 3, OTU 345, OTU 339, OTU 854, OTU 969), *Actinomyces* (OTU 1102, OTU 722), *Granulicatella* (OTU 84), *Rothia* (OTU 1179), *Fusobacterium* (OTU 707), *Leptotrichia* (OTU 156, OTU 853, OTU 589, OTU 1101), *Atopobium* (OTU 1119), *Campylobacter* (OTU 404, OTU 1299), *Veillonella* (OTU 1206), *Phyllobacterium* (OTU 488), *Acinetobacter* (OTU 860, OTU 928, OTU 943, OTU 1122), *Corynebacterium* (OTU 683), *Lactobacillus* (OTU 242), *Blautia* (OTU 1174), *Lactobacillus* (OTU 1004), unclassified *Enterobacteriaceae* (OTU 728), *Peptostreptococcaceae incertae sedis* (OTU 220), *Stenotrophomonas* (OTU 521, OTU 537), *Achromobacter* (OTU 540, OTU 682), *Delftia* (OTU 1041), *Aeromonas* (OTU 953) and *Ralstonia* (OTU 884) were all higher in RA and DM compared to N, whereas *Lachnospiraceae incertae sedis* (OTU 1294), *Pseudobutyrivibrio* (OTU 957), *Veillonella* (OTU 783), *Corynebacterium* (OTU 683), *Leptotrichia* (OTU 1101), *Lactobacillus* (OTU 242), *Blautia* (OTU 1174), unclassified *Enterobacteriaceae* (OTU 728), *Leptotrichia* (OTU 589), *Peptostreptococcaceae incertae sedis* (OTU 220), *Stenotrophomonas* (OTU 521, OTU 537), *Acinetobacter* (OTU 860) and *Brevundimonas* (OTU 916) were all lower in RA and DM than in N. Between RA and DM, *Lachnospiraceae incertae sedis* (OTU 1294), *Prevotella* (OTU 345, OTU 854), *Leptotrichia* (OTU 156, OTU 589, OTU 853), *Atopobium* (OTU 1119), *Campylobacter* (OTU 404), *Acinetobacter* (OTU 928), *Corynebacterium* (OTU 683), *Blautia* (OTU 1174), *Lactobacillus* (OTU 1004), *Stenotrophomonas* (OTU 521), *Achromobacter* (OTU 540), *Aeromonas* (OTU 682, OTU 953), *Delftia* (OTU 1041) and *Acinetobacter* (OTU 943, OTU 1122) were lower in RA than in DM, and other genera in 41 OTUs were higher than in DM (Fig. 5).

### Crucial bacteria of the lung microbial communities in RA, DM and healthy control groups

**Fig. 6:**
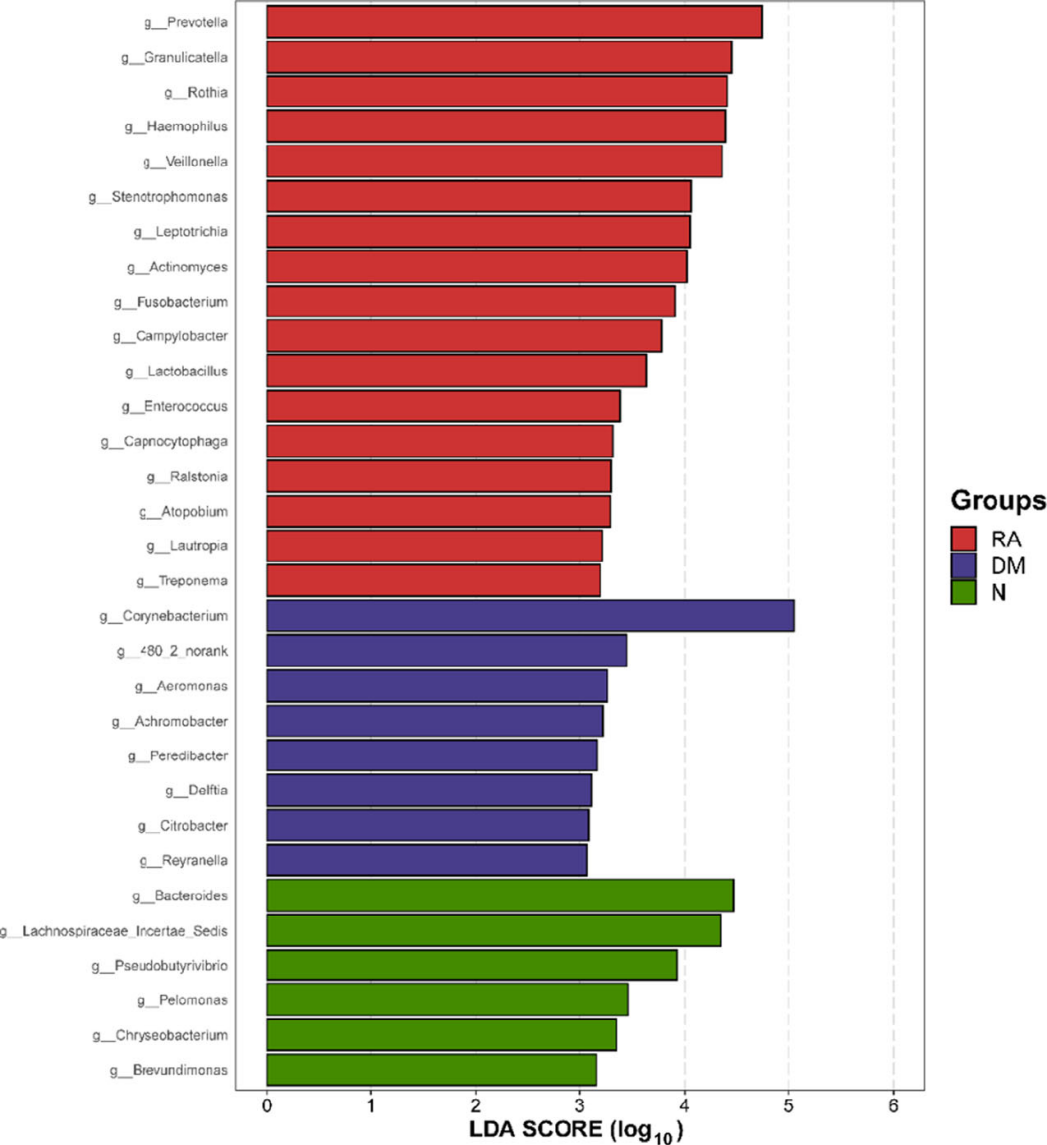
LEfSe and LDA analyses based on OTU characterizations of lung microbiota in RA (*n* = 19), DM (*n* = 7) and N (*n* = 18). Histogram of LDA scores calculated for selected taxa showing a significant difference in microbe type and abundance among RA (red), DM (blue) and N (green). LDA score on the log_10_ scale is indicated at the bottom. The significance of the microbial marker increases with the LDA score.

An LEfSe analysis and the linear discriminant analysis (LDA) genus score (Fig. 6) confirmed that 31 microbial biomarkers were clearly distinguished among RA, DM and H. Moreover, the divergence among groups was highly significant (*P* < 0.05). Biomarker names, LDA scores, log values and *P*‐values are provided in the Supporting information.

The LDA scores in Fig. 6 show significant differences in bacterial genera present among RA, DM and N. Seventeen genera including *Prevotella*, *Granulicatella*, *Rothia*, *Haemophilus, Veillonella*, *Stenotrophomonas*, *Leptotrichia* and *Actinomyces* predominated in the RA group (*P* < 0.05; LDA > 3). Eight genera including *Corynebacterium*, *480_2_norank*, *Aeromonas* and *Achromobacter* predominated in the N (*P* < 0.05; LDA > 3). Six genera including *Bacteroides*, *Lachnospiraceae incertae sedis, Pseudobutyrivibrio* and *Pelomonas* predominated in the N group (*P* < 0.05; LDA > 3).

### Gene function analysis in different groups

**Fig. 7:**
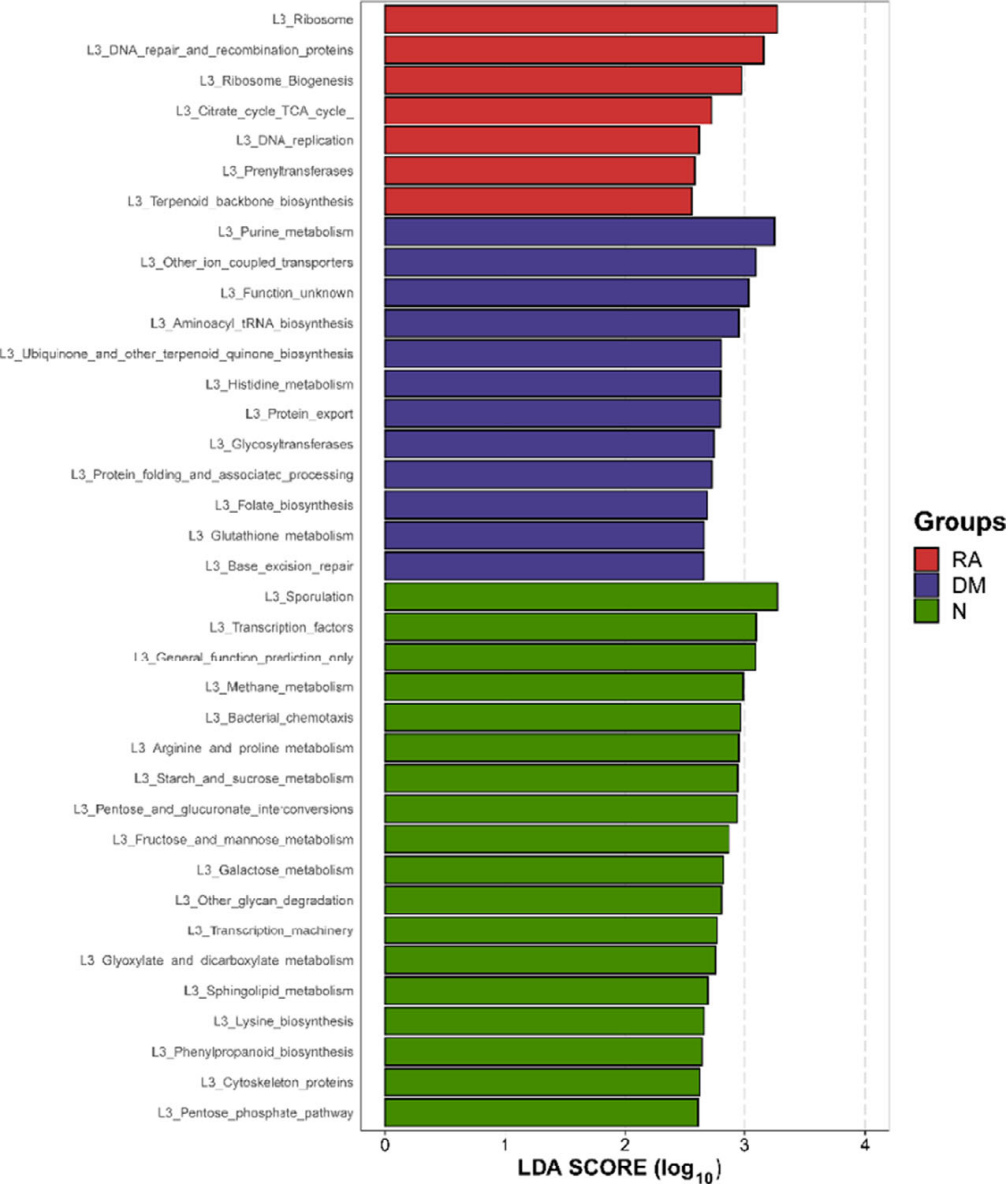
Functional analysis of predicted 16S rRNA data. Differentially abundant KEGG pathways among RA (*n* = 19), DM (*n* = 7) and N (*n* = 18) identified by LEfSe. Histogram of LDA scores calculated for selected pathways showing a significant difference in gene functions among RA (red), DM (blue) and N (green). The LDA score on the log_10_ scale is indicated at the bottom. The significance of the microbial marker increases with the LDA score.

To elucidate the functional and metabolic alterations of the lung microbiomes among the RA, DM and N groups, the gene function prediction was inferred from the 16S rRNA data, and the functional potential of the lung microbiota was analyzed using picrust. The differentially abundant KEGG pathways among the RA, DM and N groups were identified by LEfSe (Fig. 7). Biomarker names, LDA scores, log values and *P*‐values are provided in the Supporting information. Seven KEGG pathways including ribosome, DNA repair and recombination proteins, and ribosome biogenesis were enriched in RA (*P* < 0.05, LDA > 2.5). Twelve pathways including purine metabolism, other ion coupled transporters and aminoacyl tRNA biosynthesis were enriched in DM (*P* < 0.05, LDA > 2.5). Seventeen pathways including sporulation L3 transcription factors, general function prediction only, methane metabolism, bacterial chemotaxis, arginine and proline metabolism, starch and sucrose metabolism, and pentose and glucuronate interconversions were enriched in the N group.

## Discussion

Microbial–immune cell interactions can form an adaptive immune response in hosts, and the contribution of microorganisms to the pathogenesis of autoimmune diseases is credible and possible. Recently, an increasing number of studies have used 16S rRNA gene pyrosequencing technology to explore the lower and upper respiratory tract microbiota in health and disease [26, 27]. A deeper understanding of the role of lung microbiota as a mediator of inflammation has recently emerged. Microbes including viruses, bacteria and environmental fungi have long been hypothesized to play a role in the pathogenesis of ILDs. The findings from novel relevant studies have revealed that the human distal airways harbor several bacterial species constituting a unique ecological community [28] and also that an increased bacterial burden and/or an abundance of potentially pathogenic bacteria are associated with disease progression, acute exacerbations and mortality in idiopathic pulmonary fibrosis [19, 29, 30]. A recent study also investigated the lung microbiota composition in patients with early rheumatoid arthritis and found reduced bacterial species diversity compared to controls [20]. The present study demonstrates that lung microbiota may play a different role in inflammatory lung disease.

As a highly progressive disease, management of DM‐ILD remains a challenge. Although DM‐ILD etiologies include environmental factors such as infection, malignancy and drug toxicities from statins or immune checkpoint inhibitors [31], their role in the pathogenesis of DM‐ILD initiation or progression remains poorly understood. To our knowledge, the present study is the first attempt to investigate the lung microbiome in this disease.

In our study, we profiled the differences in lung microbiota of RA, DM and healthy controls using 16S rRNA high‐throughput sequencing. We showed that, compared to healthy controls, the alpha diversity in terms of both the evenness and richness is higher in patients with DM and RA, although there were no differences between DM and RA. As previously reported, the α‐diversity of the lung microbiome in RA was decreased compared to healthy controls [20], and other research on mucosal sites has reported similar findings [32, 33, 34]. However, our results conflicted with these findings, possibly because bacteria dysbiosis caused a decline in lung function, which increased the communication between the lung and the environment. Furthermore, more taxa occupied the lung’s ecological niche, and these taxa supplied a much more suitable environment for pathogenic bacteria. However, much more research is needed on this phenomenon. Importantly, the increased taxa were not shared among RA, DM and healthy controls. Indeed, some of the shared taxa between RA and DM had a different abundance, such as the genus *Prevotella,* which was decreased in DM. Some species of *Prevotella*, such as *Prevotella copri*, were considered pathogenic [35], and various species belonging to the *Prevotella* genus possess a different functional potential and therefore impact the clinical outcome differentially [35], including the generation of a 27‐kDa protein by DR‐presentation from *P. copri*, which could stimulate T‐helper‐cell 1 immune responses [36] and suppressed arthritis in humanized HLA‐DQ8 mice [37]. The *Streptococcus* genus was more abundant in samples from lung tissues and bronchoscopy in patients with cancer [38]. In the present study, there was no significant difference in Streptofocus between RA, DM and healthy controls, which may potentiate cancerous processes. *Corynebacterium* is a common species in the skin microbial community [39], acting as the key stabilizer taxon or commensal in healthy skin, and this species might be replaced or inhibited once the competing network is disrupted by other pathogens [40]. Here, we saw more *Corynebacterium* in DM than in RA and healthy controls, and these findings in line with prior studies [40]. The taxa that we observed in RA, DM and healthy controls were similar to those reported previously, and there may be many unknown functions for each taxon [40]. Although the present study predicts the function of the lung microbiota, validation studies are still needed. A combination of descriptive and hypothesis‐driven research is essential for identifying potential microbial targets preclinically and establishing the treatment of autoimmune diseases.

The main limitation of the present study is the relatively small sample size. Our preliminary findings require confirmation in larger studies. Importantly, our study revealed the differences in lung microbiota and prediction functions among RA, DM and healthy controls. We did not collect data on factors such as living environment, lifestyle and dietary habits, which can cause changes in lung microbiota. Therefore, a correlation between microbiota and such factors is lacking. Also, we knew that the greater the number of samples collected, the better the statistical power of the study. However, BALF sample collection was difficult to implement. Because bronchoscopy is invasive, patients with RA or DM are not willing to undergo bronchoalveolar lavage if there is no obvious lung CT abnormality; accordingly, it is difficult to include RA or DM patients without interstitial lung disease as control participants. To better elucidate the causal relationship between the occurrence of DM and RA with the lung microbiota, samples will be collected longitudinally for subsequent in‐depth studies, which will shed further light on the mechanisms of autoimmune disease development.

In conclusion, we profiled the differences in the lung microbiota of patients with RA, DM and healthy controls using 16S rRNA high‐throughput sequencing. Our data demonstrate that the α‐diversity of lung microbiota in patients with DM and RA was higher than that of healthy controls, although there was no difference between DM and RA. However, comparisons of DM and RA showed much more varied genera, with 14 genera more prevalent in DM than in RA, such as *Lachnospiraceae incertae sedis*, *Prevotella*, *Leptotrichia*, *Atopobium*, *Campylobacter*, *Acinetobacter*, *Corynebacterium*, *Blautia*, *Lactobacillus*, *Stenotrophomonas*, *Achromobacter*, *Aeromonas*, *Delftia* and *Acinetobacter*. As the differences in microbiota between DM and RA based on LEfSe suggest, we cannot discount the role of these bacteria in lung disease. Gene function analysis has shown that DM and RA have different metabolic pathways. Although we investigated lung microbiota and gene function prediction, we note that the present study only described lung microbiota in patients with RA and DM, and therefore causality cannot be inferred. However, further studies are needed to elucidate the role of the lung microbiota and its potential association with lung disease. Research on the lung microbiome and lung disease may also open new opportunities for developing biomarkers to identify high‐risk patients.

## Conflict of interests

The authors declare that they have no conflicts of interest.

## Author contributions

Y Zheng and XM Tan conceived and designed the project. Y Zheng, YY Lou, BJ Fan, LY Zhang, XD Wang and ZW Chen acquired the data. YY Lou and Q Wei analyzed and interpreted the data. Y Zheng and YY Lou wrote the paper.

## Data Availability

Data are available from the corresponding author upon reasonable request.
